# The Importance of the Mesio-Distal Relation of the Two Arches during Childhood

**Published:** 1903-11

**Authors:** S. M. Weeks

**Affiliations:** Philadelphia


### THE IMPORTANCE OF THE MESIO-DISTAL RELATION OF THE TWO ARCHES DURING CHILDHOOD.[1]

BY DR. S. M. WEEKS, PHILADELPHIA.

I WISH to invite your attention for a few minutes this evening to a phase in the care of children's teeth which it seems to me has been neglected. We hear a great deal about the proper care of

[1] Read before the Academy of Stomatology, Philadelphia, March, 1903.

the mouths of children for the preservation of their teeth, and, when decayed, about the best methods of replacing lost structure, etc. All of this we know to be of fundamental importance, but there are other conditions equally vital.

It is to the matter of irregularities and the mesio-distal relation of the two arches to which I refer. I believe the early recognition of certain abnormal tendencies in the eruption of the teeth to be of such importance that its neglect makes us responsible for a long line of subsequent difficulties. On the other hand, a right understanding of the conditions present and those sure to follow enables us, by small effort, to correct these errors of development, which, if allowed to continue, would cause us much trouble and our patients much inconvenience.

In using the term children in this paper I wish to explain that I mean those in that period of life from the time the first permanent molars are erupted until the time when all of the teeth except the third molars are in place; in other words, that period when, if asked to give advice about an irregularity, it has been customary to say, "Oh, better leave it until all of the teeth are in place." The bugbear of procrastination has attached itself to us so long that I want to emphasize my belief that this is an error for which without injustice we could be strongly censured.

There is no doubt that in many cases, from the age of six to fourteen, we find certain irregularities beginning to be manifest concerning which it is hard to decide as to the best course to pursue. Many are cases where very little effort on our part would have set these irregularities right at this age, when, as with many which I have seen, when fully developed and fixed, two years was considered a short time in which to correct.

The time at my disposal is so out of proportion to the number of phases which should be considered that I shall hurriedly pass many, taking up more in particular one phase, the effects of which seems to be so pernicious and far-reaching as to brook no delay in receiving attention. This phase is evidenced by the loss of the proper relation between the superior and inferior arches. For a starting-point we must assume some condition which will indicate the normal relation. From the fact that the first molars are the first teeth of the permanent set to erupt, I believe we can do no better than to depend on them as a basis from which to start. If these are normal, then the two arches may be said to be correctly related to each other mesio-distally.

There are several ways in which variations from the normal may be manifested, only two of which will concern us in this paper. One is where the mesio-buccal cusps of the upper first molar occludes mesially to the mesio-buccal groove of the lower first molar. The other is just the reverse. Only, I believe, in hereditary cases is this condition noted before the eruption of the first molars; after that time it may develop at almost any stage up to the time of the fully erupted denture, and in a few cases not even excepting the third molars.

There are several groups of conditions to which we may look for causes of this abnormal relation. The first we might call a local group, because it is in conditions existing in the arrangement of the teeth themselves that we find the cause. These are many, and I can take time to enumerate only a few: Loss of a deciduous tooth, thus allowing teeth to move forward or backward, as the case may be. Loss of space in the arch through decay on the approximal surfaces. Both of these conditions make it possible for the first permanent molars to take a position anterior to that which nature intended. Too long retention of a temporary tooth, thus deflecting the permanent tooth which is to follow. Too early eruption of some of the permanent teeth. A supernumerary tooth in the arch, enlarging it beyond the normal. Loss of a permanent tooth, etc. In fact, any obstruction which fails to allow the lower jaw to be occluded with the upper in its normal position.

In another group of cases we may find our cause arising from conditions outside the dental apparatus. This would be so in cases of mouth-breathing, persistent habits of thumb-sucking, lip-biting, etc.; also in some cases I believe it to be possible for the third molar erupting under certain conditions to be a cause.

In many of these cases we must look also for some change in the temporo-maxillary articulation. Were it not for some abnormality in this articulation we would not find these classes of malocclusion having such a marked effect on the physiognomy. Again, when we find, under favorable conditions, that we can in a few weeks effect a change of several millimetres in the relation between the superior and inferior arches, we feel that this must have been by the means of a change in the articulation rather than in the alveolus. There are several reasons why this phase should receive attention at the first indication of a developing abnormality.

It is in these cases that the facility of mastication is most interfered with. It is not so very rarely that we find cases where the lower jaw is drawn forward to such an extent that, in conjunction with a contracted upper arch, we find very few, often no more than four, teeth in occlusion.

Next are those many cases of altered appearance for which this condition is responsible. I believe we have small conception of the number of faces which are given an appearance of weakness because the mandible has been allowed to drop back to a point distal to the normal. On the other hand, the reverse condition, while it offers no suggestion of weakness of character, brings us no less number of facial deformities.

In closing, I wish to say just a few words relative to the correction of such cases. Of course, it would be much easier to pay attention to the early conditions, thus preventing the occurrence of any irregularity; hence, if a tooth is lost, that space occupied by it should be retained. If space has been lost by caries on the approximal surfaces, it should be regained and retained by the use of gutta-percha or some other proper method. Naturally, whatever be the cause it should be removed. Of course, there are many cases that we as individuals do not see in time to treat in this early stage. While it is essential that all cases of irregularities should be treated as soon as brought to our notice, it is doubly true of those where the normal mesio-distal relation of the two arches is being lost. It is then that the loss of facial harmony is being effected, and we have another factor entering, which in importance is perhaps second to none.

In cases of this type it has sometimes seemed easy to extract teeth and correct a prognathism, but this procedure does nothing towards correcting abnormalities in the facial lines. I believe that there are but a *very* few of these where extracting should be countenanced. The teeth should be placed in their proper alignment and the arches restored to their normal occlusion, thereby bringing the facial lines into harmony.

